# Adolescent Physical Activity at Public Schools, Private Schools, and Homeschools, United States, 2014

**DOI:** 10.5888/pcd17.190450

**Published:** 2020-08-20

**Authors:** Calvin P. Tribby, April Oh, Frank Perna, David Berrigan

**Affiliations:** 1 Health Behaviors Research Branch, Behavioral Research Program, Division of Cancer Control and Population Sciences, National Cancer Institute, Bethesda, Maryland; 2 Cancer Prevention Fellowship Program, Division of Cancer Prevention, National Cancer Institute, Bethesda, Maryland; 3 Health Communication and Informatics Research Branch, Behavioral Research Program, Division of Cancer Control and Population Sciences, National Cancer Institute, Bethesda, Maryland

## Abstract

**Introduction:**

Physical activity overall and during school-related opportunities among homeschool adolescents are poorly documented.

**Methods:**

We used data from the National Cancer Institute’s Family Life, Activity, Sun, Health, and Eating (FLASHE) study, a national sample of parent–child dyads. We examined reported frequency of physical activity in middle-school and high-school respondents (N = 1,333). We compared the overall physical activity by school type (ie, public school, private school, and homeschool), compared school-related contexts (eg, recess, physical education [PE] class), and tested for level of physical activity by school for those reporting PE.

**Results:**

Middle-school homeschool adolescents reported less physical activity during school hours compared with public school, but not private school, adolescents. Physical activity was not different by school type for out of school or weekends. Physical activity of high-school homeschool adolescents was not different from that of high-school adolescents at traditional schools; homeschool adolescents in both middle and high school reported less physical activity in PE compared with public and private school adolescents. Other school-related contexts of physical activity were not different by school type. More homeschool students reported not having PE (middle school, 54.8%; high school, 57.5%) compared with public (middle school, 18.7%; high school, 38.0%) or private schools (middle school, 13.5%; high school, 41.5%).

**Conclusion:**

Homeschool adolescents in middle school reported less physical activity compared with middle-school adolescents in traditional schools during school hours, likely because of having fewer PE classes and less physical activity during PE.

**Summary**

**What is already known on this topic?**

There is little evidence for how adolescent physical activity differs in homeschool, public, or private school settings. Existing findings are mixed and the physical activity levels of homeschool adolescents and their opportunities for physical activity are not well documented.

**What is added by this report?**

Analysis of these data suggest that physical education classes are an important contributor to physical activity and that these classes are less prevalent in homeschool curricula.

**What are the implications for public health practice?**

Further research could better quantify whether homeschool adolescents are accumulating less physical activity than their public and private school peers. School districts and non-profits supporting homeschooling could examine support for homeschool physical education curricula.

## Introduction

Physical activity is important for adolescent health and chronic disease prevention (1), yet most US adolescents (78%) do not report meeting recommended levels of physical activity (2). The 2018 Physical Activity Guidelines for Americans recommend at least 60 minutes of moderate to vigorous physical activity (MVPA) each day for adolescents (3), and adolescent physical activity levels are strong predictors of adult physical activity levels (4). Physical activity levels for adolescents who attend different types of school (public, private, or home) are poorly understood. Furthermore, school-related opportunities for physical activity (travel to and from school and during physical education [PE] class, recess, or lunch) and associations with adolescents’ physical activity levels in the different school types are largely unknown. Traditional schools (ie, public or private) have many school-related opportunities for physical activity: before and after school and during recess, lunch, PE class, and team sports (5–7).

Research on physical activity and homeschools has described adolescents’ physical activity levels (8) and changes associated with an intervention (9). However, results are mixed as to whether there is a difference in physical activity between public school and homeschool students. Two studies reported no difference (10,11) and 1 study reported lower levels of physical activity in homeschool students (12). The homeschool students had fewer weekday steps and spent less time in MVPA compared with public school adolescents; there was no difference in weekend activity (12).

We examined overall physical activity of adolescents and physical activity levels during school-related opportunities by school type. Our first aim (Aim 1) compared physical activity levels for adolescents who attended public or private schools and homeschools during school and nonschool hours. Our second aim (Aim 2) examined differences in physical activity associated with school-related opportunities. A final adjusted analysis examined physical activity levels during PE class. For all comparisons, we hypothesized that adolescents in homeschools participate in less physical activity than adolescents in traditional school settings.

## Methods

### Sample

In this cross-sectional study, we used the Family Life, Activity, Sun, Health, and Eating (FLASHE) public use data set (13). FLASHE was a national web-based Ipsos Consumer Opinion Panel sample conducted in 2014 that fully enrolled 1,945 parent–child dyads (participation rate, 38.7%) from 5,027 dyads that were screened for eligibility (14). Respondents provided demographic information, such as age, sex, race/ethnicity, and employment status; health behaviors, such as hours of sleep, diet, and availability of fruits and vegetables; school variables of type and grade level; and details of physical activity and sedentary time, captured with the Youth Activity Profile (YAP) (13). The sample for this study was adolescents who completed the physical activity survey (n = 1,661) (14). Respondents in elementary school (n = 27), not currently in school (n = 96), in another type of school (n = 32), or missing values (school type [n = 34], age [n = 1], sex [n = 6], race/ethnicity [n = 15], self-reported weight status [n = 7] or any of the physical activity questions [n = 110]) were excluded. After these exclusions, the analytic sample included 1,333 adolescents.

### Measures

The YAP portion of the survey asked the adolescent respondents about their physical activity in the past 7 days overall, during school hours, and outside of school hours. Questions about physical activity during school hours included 1) active travel (walking or cycling) to and from school, 2) PE class, 3) recess, and 4) lunch. Questions about physical activity during nonschool hours included activities 1) before and after school, 2) on weeknights, 3) on Saturday, and 4) on Sunday. Responses to the activities before and after school and on weeknights and for active travel to and from school used the integer interval scale from 1 (“0 days [never]”) to 5 (“4-5 days [most every day]”). Responses to the activities during PE class, recess, and lunch used the ordinal scale from 0 (“didn’t have activity”) to 6 (“[running or moving] almost all of the time”). Responses to physical activity on Saturday and Sunday used the interval scale from 1 (“no activity”) to 5 (“large amounts of activity [2 hours]”). The YAP questions were validated with accelerometer data to provide acceptable estimates of daily and weekly MVPA (15). Specifically, the YAP-estimated time spent in MVPA was statistically equivalent (using the 10%–15% equivalence zone criteria) to accelerometer-measured estimates for during school hours and out of school; the agreement was lower for the weekend section (15). Sedentary time used the ordinal scale of 1 (“I spend almost none of my free time in sedentary activities”) to 5 (“I spend almost all of my free time in sedentary activities”). Weight status used the scale of 1 (“I’m very underweight”) to 3 (“My weight is just right”) to 5 (“I’m very overweight”). For parents, we used any walking of at least 10 minutes and any MVPA in the past 7 days.

We assessed whether there was variation in the Classification of Laws Associated with School Students (CLASS) regarding physical activity and physical education requirements in the sample. CLASS is an online resource (https://class.cancer.gov/) that scores the strength of state laws related to physical activity and nutrition recommendations by school level (16). We used the 2014 codes in the CLASS policy areas of physical education time requirements and physical activity time requirements as variables to assess policy comparability of the sample. PE requirements used the ordinal categories from 0 (“no codified law”) to 5 (“law requires recommended standard” [≥225 min/wk for middle- or high-school students]). Physical activity requirements (which may or may not include physical education time) were categorized from 0 (“no PA requirement or recommendation”) to 5 (“state requires school districts provide PA for a minimum of 150 minutes per week, for middle and high schools”). Each state received a separate code for each school level (elementary, middle, and high school).

### Statistical analysis

Aim 1 sought to determine any differences in physical activity levels between adolescents who attended public or private schools and homeschools. We assessed this with multivariate analysis of variance (MANOVA), testing the difference in means of physical activity by type of school for 3 periods: during school, out of school weekday, and weekend. Responses to the physical activity questions were integer, interval, or ordinal, but the ordinal variables have approximately equal numeric distance between responses, which allowed us to analyze both response types with continuous methods (17). Aim 2 sought to determine differences in reported physical activity between school types for the school-based opportunities, which were assessed with MANOVA for unadjusted differences. We used post-hoc contrasts to test means between public or private and homeschools to limit the number of comparisons and because we were not interested in testing between public and private schools.

For the adjusted analysis of school type on physical activity during physical education class, the subset of respondents (n = 909) who reported having PE class was examined with logistic regression. Based on the loss of sample, these analyses were exploratory. The dependent variable was a dichotomous variable of at or above the median physical activity level during PE class, which was “almost all the time” for both middle and high schools; the reference category was below the median level. We recoded the 5-category ordinal physical activity level into a dichotomous variable, because some categories had no observations across independent variables and school type (eg, sex). The independent variables were school type (public, private, or homeschool [reference]), sex (male [reference]), and age (years). All statistical tests were performed using SAS version 9.4 (SAS Institute, Inc) and were stratified by school level (middle and high school). Differences were significant at *P* < .05.

FLASHE received approval from the Office of Management and Budget, the National Institutes of Health institutional review board, and the Westat institutional review board. This study analyzed the publicly available deidentified data set and was exempt from review.

## Results

We found differences in the demographic and weight status characteristics of students, although none were significant. For middle schools, 39% of homeschool adolescents were female, compared with 52% of public and 59% of private school adolescents (*P* = *.*24) (Table 1). Homeschool adolescents were more likely to be Hispanic (19.4%) and less likely to be non-Hispanic Black or African American (6.5%) or other races/ethnicities (6.5%) compared with public or private school adolescents (*P* = *.*38). More homeschool adolescents reported their weight was just right (71.0%), compared with public (61.6%) and private (59.5%) school adolescents (*P* = *.*91).

**Table 1:** Percentages of Selected Characteristics of Surveyed Middle-School and High-School Students and Their Parents, FLASHE Study, United States, 2014

Characteristic	Middle-School Students				High-School Students			
	Public School (N = 460)	Private School (N = 37)	Homeschool (N = 31)	*P *Value^a^	Public School (N = 692)	Private School (N = 65)	Homeschool (N = 40)	*P *Value^a^
**Female sex**	52.0	59.5	38.7	.24	51.5	50.8	50.0	.98
**Race/ethnicity**								
White, non-Hispanic	62.7	67.6	67.7	.38	65.3	70.8	72.5	.96
Black or African American, non-Hispanic	16.7	10.8	6.5		16.9	13.9	12.5	
Hispanic	9.1	8.1	19.4		10.7	7.7	7.5	
Other, non-Hispanic	11.5	13.5	6.5		7.2	7.2	7.5	
**Weight status^b^**								
Underweight	12.9	13.5	9.7	.91	9.1	6.1	12.5	.30
Just right	61.6	59.5	71.0		61.8	63.1	72.5	
Overweight	25.4	27.0	19.4		29.1	30.8	15.0	
**Any hours worked for employment**	6.2	0	16.2	.03	19.3	23.1	10.0	.24
**Parent’s PA in last 7 days**								
Any moderate to vigorous activity	77.4	75.7	67.7	.46	76.8	81.5	82.5	.59
At least 10 min of walking	79.5	64.9	77.4	.11	77.7	75.4	85.0	.50
**PE and PA state-level laws^c^**								
Any PE time requirements	99.8	97.3	100	.24	99.4	100	100	.99
Any PA time recommendations or requirements	57.1	54.1	51.6	.78	46.4	41.5	42.5	.60

Abbreviations: CLASS, Classification of Laws Associated with School Students; FLASHE, Family Life, Activity, Sun, Health, and Eating; PA, physical activity; PE, physical education.

a *P* values determined by using Fisher exact χ^2^ tests.

b Participants rated their current weight as underweight (“I’m very underweight” or “I’m slightly underweight”), just right (“my weight is just right”), or overweight (“I’m slightly overweight” or “I’m very overweight”).

c School level–specific state laws; state laws generally do not apply to private and homeschools. Source: CLASS database (16).

High-school adolescents were more likely to report being overweight at public (29.1%) or private (30.8%) schools compared with homeschools (15.0%) (*P* = *.*30) (Table 1). Most adolescents lived in states with PE recommendations or requirements. Any walking and any MVPA was not different between school types for high-school or middle-school parents.

Middle-school students at private schools were younger than middle-school homeschool students (*P* < *.*05). For high school, homeschool students were more sedentary than public school students (*P* < *.*05), and public school students got less sleep, 7.9 hours (95% confidence interval [CI], 7.7–8.1) compared with homeschool students, 9.0 hours (95% CI, 8.6–9.4) (*P* < *.*05) (Table 2).

**Table 2:** Means and 95% Confidence Intervals of Select Characteristics of Surveyed Middle-School and High-School Students, FLASHE Study, United States, 2014

Variable	Middle-School Students			High-School Students		
	Public School (N = 460)	Private School (N = 37)	Homeschool (N = 31)	Public School (N = 692)	Private School (N = 65)	Homeschool (N = 40)
Age, y	13.0 (12.9-13.1)	12.6^a^ (12.4-12.8)	13.2^a^ (12.9-13.5)	15.6 (15.5-15.7)	15.3 (15.0-15.6)	15.7 (15.4-16.0)
Sedentary time^b^	3.0 (2.9-3.1)	2.6 (2.3-2.9)	3.0 (2.6-3.4)	3.0^a^ (2.9-3.1)	3.1 (2.9-3.3)	3.5^a^ (3.3-3.8)
Hours of weekday sleep	8.5 (8.2-8.8)	9.0 (8.7-9.3)	9.4 (8.1-10.7)	7.9^a^ (7.7-8.1)	8.2 (7.9-8.5)	9.0^a^ (8.6-9.4)

Abbreviation: FLASHE, Family Life, Activity, Sun, Health, and Eating.

a Significant at *P* < *.*05 between school types (public vs home and private vs home); determined by using Tukey’s Studentized Range Test.

b Composite score from the Youth Activity Profile for outside-of-school sedentary activity; includes items of reported television time, video game time, computer time, telephone/text time, and overall sedentary habits over the past 7 days. Responses ranged from 1 (didn’t do activity) to 5 (≥3 h/d). Composite score is an equally weighted average of items.

### Unadjusted analysis

**Figure 1:**
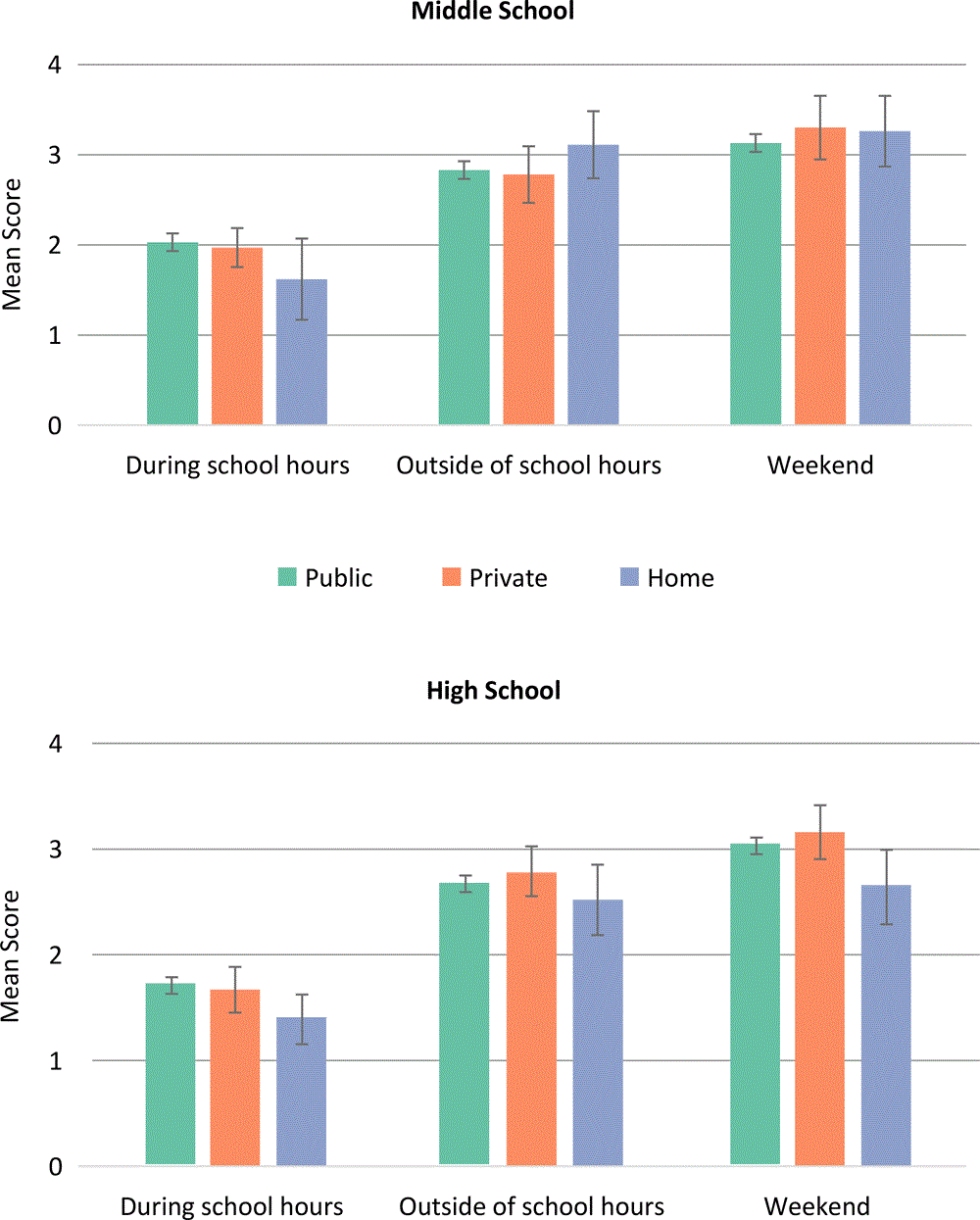
Physical activity mean scores (95% confidence interval), by school type, for 3 contexts: during school hours, weekday outside of school hours, and weekends, stratified by school level, FLASHE Study, 2014. During school hours was the mean score of activity to and from school, during PE class, during recess, and during lunch (scored from 0, “didn’t have activity,” to 6, “[running or moving] almost all of the time”). Outside of school hours was the mean score of activities outside of school: before and after school and activity on weeknights (scored from 0 [never] to 5 [4 to 5 days or most every day]). Weekend was the mean score of activity on Saturday and Sunday (scored from 1 [no activity] to 5 [large amounts of activity, 2 hours of activity]). Values are mean (95% confidence interval); bars indicate confidence intervals. Post-hoc contrast between middle-school, public-school, and homeschool students, *P* = .03. Abbreviation: FLASHE, Family Life, Activity, Sun, Health, and Eating; PE, physical education.

The one significant difference for mean physical activity score by school type was for middle-school students during school hours: public school, 2.0 (95% CI, 1.9–2.1) and homeschool, 1.6 (1.2–2.1) (*P* = *.*02) (Figure 1). The students were comparable across school type for physical activity outside of school hours on weekdays and on weekends. For students in all schools, physical activity was higher during these periods, compared with during school hours.

**Table:** 

School type	During School Hours	Outside of School Hours	Weekend
	Physical Activity Level, Mean Score (95% Confidence Interval)		
**Middle school**			
Public	2.0 (1.9–2.1)	2.8 (2.7–2.9)	3.1 (3.0–3.2)
Private	2.0 (1.8–2.2)	2.8 (2.5–3.1)	3.3 (2.9–3.7)
Home	1.6 (1.2–2.1)	3.1 (2.7–3.5)	3.3 (2.9–3.7)
**High school**			
Public	1.7 (1.6–1.8)	2.7 (2.6–2.7)	3.0 (3.0–3.1)
Private	1.7 (1.5–1.9)	2.8 (2.5–3.0)	3.2 (2.9–3.4)
Home	1.4 (1.2–1.6)	2.5 (2.2–2.9)	2.7 (2.3–3.0)

**Figure 2:**
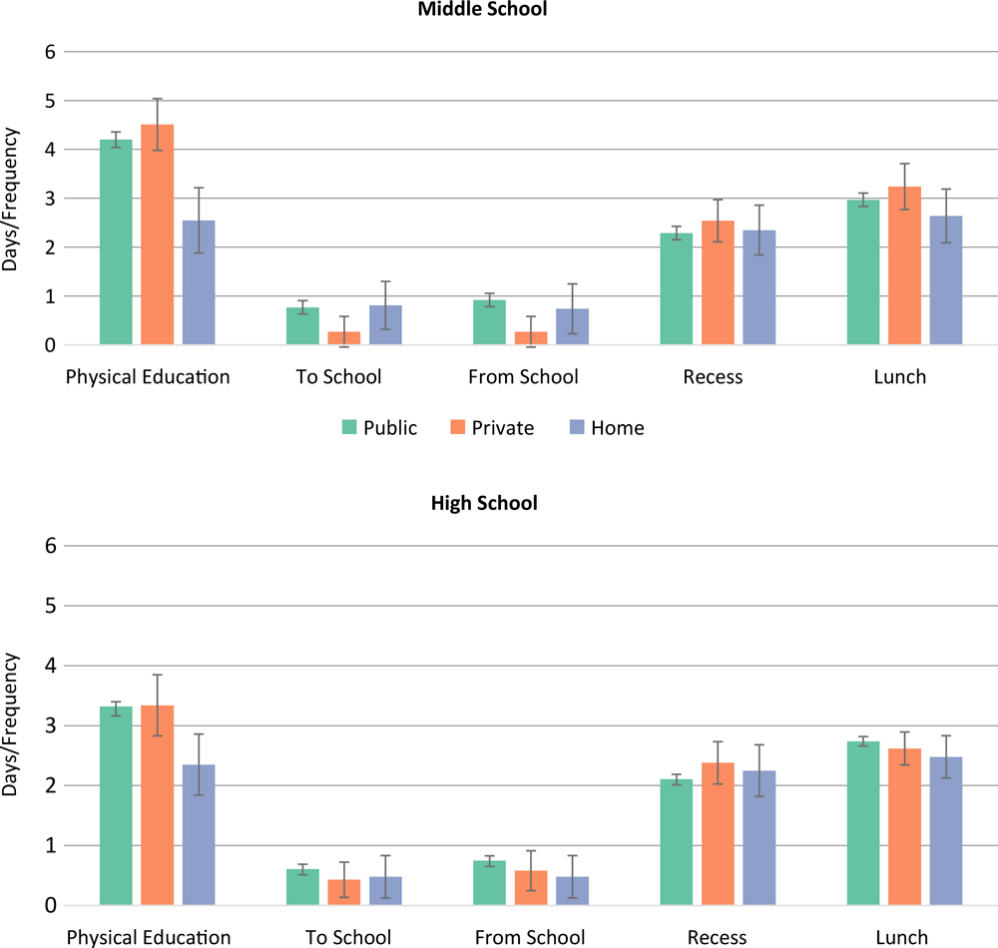
Physical activity means (95% confidence interval) by school type for the 5 during school hours contexts, FLASHE, 2014. Days of physical activity per week while traveling to school and from school, and frequency of physical activity level during physical education class, recess, and lunch. Bars indicate confidence intervals. Abbreviation: FLASHE, Family Life, Activity, Sun, Health, and Eating.

For middle-school students, there was a significant difference for physical activity in PE class at public schools (mean score, 4.2 [95% CI, 4.0–4.4]) compared with homeschool (mean score, 2.6 [95% CI, 1.9–3.2]) (*P* < *.*001) (Figure 2). Similarly, adolescents at private schools had a higher mean score of 4.5 (95% CI, 4.0–5.0) compared with homeschool adolescents (*P* < *.*001). For middle-school adolescents, the means of physical activity were not different by school type for the other school-related opportunities of travel to school, travel from school, recess, or lunch.

**Table:** 

Activity Location	Public School	Private School	Home
	Mean Days of Physical Activity (95% Confidence Interval)		
**Middle school**			
Physical education	4.2 (4.0–4.4)	4.5 (4.0–5.0)	2.6 (1.9–3.2)
To school	0.8 (0.6–0.9)	0.3 (0.0–0.6)	0.8 (0.3–1.3)
From school	0.9 (0.8–1.1)	0.3 (0.0–0.6)	0.7 (0.2–1.2)
Recess	2.3 (2.2–2.4)	2.5 (2.1–3.0)	2.4 (1.8–2.9)
Lunch	3.0 (2.8–3.1)	3.2 (2.8–3.7)	2.6 (2.1–3.2)
**High school**			
Physical education	3.3 (3.2–3.5)	3.3 (2.8–3.8)	2.4 (1.8–2.9)
To school	0.6 (0.5–0.7)	0.4 (0.1–0.7)	0.5 (0.1–0.8)
From school	0.8 (0.7–0.8)	0.6 (0.2–0.9)	0.5 (0.1–0.8)
Recess	2.1 (2.0–2.2)	2.4 (2.0–2.7)	2.3 (1.8–2.7)
Lunch	2.7 (2.7–2.8)	2.6 (2.3–2.9)	2.5 (2.1–2.8)

For high schools, adolescents at public schools had a mean score of 3.3 (95% CI, 3.2–3.5) for physical activity in PE, compared with a mean score 2.4 (1.8–2.9) for homeschool adolescents (*P* = *.*003) (Figure 2). Similarly, adolescents at private schools had a higher mean score (3.3 [95% CI, 2.8–3.8]), compared with homeschool adolescents (*P* = *.*02). For high-school adolescents, the means of physical activity were not different by school type for the other school-related opportunities of travel to school, travel from school, recess, or lunch.

The differences in physical activity in PE class were mainly from homeschool students, who reported they did not have PE class (middle school, 54.8%; high school, 57.5%) compared with public (middle school, 18.7%; high school, 38.0%) or private (middle school, 13.5%; high school, 41.5%).

### Adjusted analysis

For a subset of adolescents who had PE class, we examined physical activity levels during class (Table 3). School type and covariates were not significant for middle schools. School type was significant for high-school students who reported having physical education class (*P* = *.*003). For high school, the adjusted odds of higher physical activity levels for public-school adolescents was 5.5 (95% CI, 1.7–17.4) and for private-school adolescents was 10.5 (95% CI, 2.7–41.1) times higher compared with homeschool adolescents. Sex was also significant (*P* < *.*001); the odds of higher physical activity level during physical education class was 0.4 (95% CI, 0.3–0.6) times lower for female adolescents compared with male adolescents.

**Table 3:** Adjusted Odds and 95% Confidence Intervals of Meeting or Exceeding Median Reported Physical Activity Levels in Physical Education Class, for Adolescents Who Reported Having a Physical Education Class, FLASHE Study, United States, 2014

Variable	Middle School (n = 423)		High School (n = 486)	
	Adjusted Odds Ratio (95% Confidence Interval)	*P* Value	Adjusted Odds Ratio (95% Confidence Interval)	*P* Value
**School type**				
Home	1 [Reference]			
Public	2.1 (0.7–6.2)	.30	5.5 (1.7–17.4)	.003
Private	2.8 (0.7–10.8)		10.5 (2.7–41.1)	
**Sex**				
Male	1 [Reference]			
Female	0.7 (0.4–1.1)	.14	0.4 (0.3–0.6)	<.001
**Age**	0.8 (0.7–1.1)	.17	1.1 (0.9–1.2)	.58

Abbreviation: FLASHE, Family Life, Activity, Sun, Health, and Eating.

## Discussion

For middle school, homeschool adolescents reported less physical activity during school hours compared with public school adolescents. The overall difference in physical activity levels during school hours was likely due to lack of PE class. Studies to date of physical activity in private and homeschool adolescents have modest sample sizes, occur in a specific geographic locale, and do not include details about school-related opportunities for physical activity. Our findings suggest that homeschool students in middle and high school may be less likely to have PE class compared with public and private school students.

The main difference in physical activity was due to many homeschool students reporting not attending a PE class. Previous research has postulated that differences may be due to physical activity patterns during the day, such as not having recess or afterschool physical activity opportunities (12). We found that middle-school adolescents who were homeschooled reported less physical activity during school, which is consistent with one previous study that found that homeschool students had lower levels of physical activity compared with public school students (12). Specifically, levels of physical activity during PE class were lower for high-school homeschool students, compared with public or private school students. For middle-school students, there was not a significant association, because an equal number (n = 7) of homeschool students were at or above the median (“a lot of the time” or “almost all of the time” of physical activity level during physical education class) as were below the median. As for other physical activity opportunities during school hours, there were no significant differences by school type for active travel to and from school, recess, or lunch time. Finally, all students’ physical activity was comparable in the domains of weekday out of school hours and weekends.

In this national sample, we found that PE recommendations or requirements were similar for adolescents by school type. However, state-level policies are not generally applicable to private and homeschools, because these schooling contexts may not be required to comply with these policies. Furthermore, measures of state-level policies can be problematic at public schools owing to variations in how school districts and individual schools implement these policies (18). However, state-level policies may be a proxy for the awareness in a state of the importance of adolescent physical activity and may still influence private and homeschool practices. The physical activity recommendations or requirements outside of PE class are much more varied; however, our sample was too small to explore the association of strength of law with physical activity by school type.

Researchers have advised that private schools should employ PE specialists and follow recommended physical activity guidelines for adolescents to increase physical activity levels (19). Similar recommendations could be extended to homeschools, with considerations about how to share PE facilities and resources. Several additional approaches to increasing physical activity among homeschool students can be found in the recent literature. First, use of technology and social media have been widely explored as tools to increase physical activity. Examples include physically active video games, such as indoor games (eg, Kinect Adventures! Xbox 360; Just Dance) (20,21), outdoor games (eg, Pokemon Go) (22,23), and use of social media channels for promotion or as a source of training and guidance (eg, Facebook, YouTube) (24,25). Second and conversely, recommendations to reduce sedentary screen time overall (26,27) or breaking up screen time with physical activity breaks (28,29) may increase students’ physical activity levels. Third, promotion of adolescent physical activity benefits to parents and adolescents through resources like Move Your Way (https://health.gov/moveyourway) may help build family support for increasing physical activity (30). Finally, the promotion and activation of neighborhood parks (31,32), or state and national parks, forests, and trails (33) with resources like Discover the Forest (discovertheforest.org) and the National Parks Every Kid Outdoors Program (https://everykidoutdoors.gov/) can be strategies to increase homeschool student physical activity.

More research is needed into how shared-use agreements may leverage community resources, such as recreation centers or public pools, to facilitate homeschool PE (34). Similarly, research is needed to assess the participation rate of homeschool students in existing programs for PE classes (eg, YMCA locations that offer PE classes [35,36]) and potential barriers (eg, cost, distance). Finally, more research is needed into the dissemination of tailored physical activity guidelines and curricular activities for homeschools to meet the guidelines. Public health partnerships with national home school associations could be a start to fostering a physical activity module for the homeschool curricula.

### Limitations

Our study had several limitations. The cross-sectional nature limited our analyses to correlational and prevented inference about school type and adolescent physical activity. The limited sample size is also an issue of many homeschool physical activity studies. In the FLASHE sample, 5.3% of students were homeschooled, whereas the nationally representative prevalence of homeschool students is 3.3% across all grade levels (37). FLASHE contains a larger sample of homeschool students compared with the national prevalence, which may have improved our chance of understanding homeschool student physical activity compared with public or private school students from this sample. Conversely, the national prevalence of private school students is 9.8% and was 7.7% in our sample (37). We sought a data set that had large sample of adolescents, was national in coverage, and asked respondents specifically when physical activity occurred during the school day and on weekends. Participants in our study had higher family socioeconomic status and a larger percentage of parents who were predominantly female and non-Hispanic white compared with the US population; these differences may have resulted from data being drawn from FLASHE, an internet panel sample (14). 

For measures of the policy context, we used state-level policies stipulating public school PE and physical activity requirements (38). Stronger legislation is associated with an increase in levels of student physical activity in public schools (39). However, how policies are implemented by school districts and individual schools can mediate their effectiveness (18). Additionally, private schools and homeschools are not generally covered by state-level laws. This factor is important to consider, because private schools comprise 9.8% (2013) and homeschools comprise 3.4% (2012) of the US primary and secondary student population, for a combined total estimate of 7,169,000 students (37).

The FLASHE questions allowed identification of frequency differences in physical activity between school type. However, our analysis did not estimate minutes at different intensities (ie, moderate to vigorous) associated with specific activities. Therefore, we were unable to characterize the magnitude of the difference in physical activity levels between students and whether this difference may be detrimental to health outcomes. It is possible that, although there are differences in reported frequency of PE, for example, the results may be different when duration and intensity of physical activity are assessed. Future research can address this limitation by using activity monitors to collect physical activity intensity and duration in addition to surveys to collect the contextual settings of physical activity with a national sample.

The type of school may not entirely explain the differences in reported physical activity. For example, although we compared sex, age, race/ethnicity, sedentary time, and reported weight status between types of schools, we were not able to include all covariates in an adjusted model because of the limited sample size of homeschool adolescents. This limitation may have resulted in some confounding in our reported differences of physical activity. Additionally, lack of school environmental supports (eg, gymnasiums, equipment) may influence whether PE is offered and the level of physical activity in classes. Lastly, although one study has examined an intervention to increase physical activity in homeschool adolescents (9), further research is needed to determine whether implementation of PE classes in a large and more diverse sample of homeschools increases students’ physical activity levels.

This study contributes to research on school type and adolescents’ physical activity and suggests that many homeschool students may not be receiving the same PE or physical activity during class as do public or private school students. However, PE and physical activity findings among homeschool students are mixed, likely because of difficulty in attaining robust sample sizes for this unique population, and this continues to be an under-researched area, one that warrants dedicated investigation.
